# P052. Patient's subjective judgement on the ease of use, speed of onset of action, efficacy and tolerability of Diclofenac sodium HPβCD (25 mg/mL) solution for subcutaneous injection vs Diclofenac potassium 50 mg for oral solution for the acute treatment of migraine

**DOI:** 10.1186/1129-2377-16-S1-A164

**Published:** 2015-09-28

**Authors:** Maurizio Evangelista, Francesco Maria Piseddu

**Affiliations:** Istituto di Anestesiologia, Rianimazione e Terapia del Dolore, Università Cattolica del Sacro Cuore/CIC, UO Terapia del Dolore-Centro Cefalee UCSC/CIC, Rome, Italy; Istituto di Anestesiologia, Rianimazione e Terapia del Dolore, Università degli Studi di Cagliari, Rianimazione e Terapia del Dolore, Cagliari, Italy

## Background

Several non-steroidal anti-inflammatory drugs (NSAIDs) have been reported to be effective in the management of acute migraine: among them, Diclofenac (DF) is one of the most effective compounds when administered either orally or intramuscularly. DF is a potent NSAID that has been in clinical use for many years, particularly effective for the treatment of inflammatory, degenerative and rheumatic diseases, soft-tissue rheumatism and non-rheumatic painful and inflammatory conditions (post-traumatic, postoperative pain and migraine headache). Among the different forms of administration a novel injectable DF formulation (dissolved in a volume of 1 ml) containing an enhancer of solubility, based idrissipropilbetacyclodextrin (HPβCD) has been recently introduced in Italy. This formulation allows the use into subcutaneous (s.c.) tissue and is characterized by a bioavailability and a safety profile comparable both to the i.m. DF formulation at a dose of 75 mg/3ml (on the market for parenteral treatment) and os DF formulation at a dose of 50 mg. Up to now no study has been performed on DF to assess the point of view of patients in relation to the ease of use of a route of administration with respect to another: this contribution is the first to do so.

## Patients and methods

Setting Area: UO Terapia del Dolore-Centro Cefalee SISC UCSC/CIC. Patients (53: 35 F/18 M) with an established migraine diagnosis (according to the ICHD-III criteria), a disease duration of at least 1 year and two to six migraine attacks per month over the previous 3 months, using DF potassium (50 mg) oral solution for the acute (moderate) treatment, were switched to subcutaneous injection of DFHPβCD (25 mg). At the following visit, patients were required to compare, in addition to the parameters of speed of onset of action, efficacy and tolerability, also the ease of use of the two routes of administration.

## Results

The results are reported in Table 1.

**Table 1:** Table 1

Patient's (53: 35 F, 18 M) subjective judgement (point of view) about Diclofenac HPβCD 25 mg s.c. vs Diclofenac potassium 50 mg os	Ease of use	Speed of onset of action	Efficacy	Tolerability
	Easier Tot 48/53 F 32/35 M 16/18	Faster Tot 50/53 F 33/35 M 17/18	Better Tot 48/53 F 33/35 M 15/18	Better Tot 51/53 F 35/351 M 16/18
Most frequent provided reasons	No need vessel and water	Time to attack resolution lower	1 Better working ability 2 Lower # second drug dose 3 light and noise sensitivity reduced	No adverse reaction

## Conclusions

Patients consistently expressed a clear preference for DF HPβCD (25 mg s.c) over DF potassium (50 mg os). Since patients are treated on an individual basis, the more important question is not only which drug is best in relation to the other but, whether the chosen one has a route of administration that better fits the outcome desired by the patient, encompassing also ease of use and feelings of well-being on an individual basis and by the healthcare provider. Compared with the DF potassium (50 mg os) reference therapy, DF HPβCD (25 mg s.c) presents interesting advantages in terms of ease of use, onset of analgesic effect and tolerability profile. Further studies are needed to confirm the data obtained from this preliminary investigation.

Written informed consent to publish was obtained from the patient(s).

